# Molecular Targets of Epigallocatechin—Gallate (EGCG): A Special Focus on Signal Transduction and Cancer

**DOI:** 10.3390/nu10121936

**Published:** 2018-12-06

**Authors:** Aide Negri, Valeria Naponelli, Federica Rizzi, Saverio Bettuzzi

**Affiliations:** 1 Department of Medicine and Surgery, University of Parma, Via Gramsci 14, 43126 Parma, Italy; aide.negri@unipr.it (A.N.); federica.rizzi@unipr.it (F.R.); saverio.bettuzzi@unipr.it (S.B.); 2 National Institute of Biostructure and Biosystems (INBB), Viale Medaglie d’Oro 305, 00136 Rome, Italy; 3 Centre for Molecular and Translational Oncology (COMT), University of Parma, Parco Area delle Scienze 11/a, 43124 Parma, Italy

**Keywords:** green tea catechins, epigallocatechin-gallate (EGCG), 67LR, cancer apoptosis, cell death, chemoprevention, gene expression

## Abstract

Green tea is a beverage that is widely consumed worldwide and is believed to exert effects on different diseases, including cancer. The major components of green tea are catechins, a family of polyphenols. Among them, epigallocatechin-gallate (EGCG) is the most abundant and biologically active. EGCG is widely studied for its anti-cancer properties. However, the cellular and molecular mechanisms explaining its action have not been completely understood, yet. EGCG is effective in vivo at micromolar concentrations, suggesting that its action is mediated by interaction with specific targets that are involved in the regulation of crucial steps of cell proliferation, survival, and metastatic spread. Recently, several proteins have been identified as EGCG direct interactors. Among them, the trans-membrane receptor 67LR has been identified as a high affinity EGCG receptor. 67LR is a master regulator of many pathways affecting cell proliferation or apoptosis, also regulating cancer stem cells (CSCs) activity. EGCG was also found to be interacting directly with Pin1, TGFR-II, and metalloproteinases (MMPs) (mainly MMP2 and MMP9), which respectively regulate EGCG-dependent inhibition of NF-kB, epithelial-mesenchimal transaction (EMT) and cellular invasion. EGCG interacts with DNA methyltransferases (DNMTs) and histone deacetylases (HDACs), which modulates epigenetic changes. The bulk of this novel knowledge provides information about the mechanisms of action of EGCG and may explain its onco-suppressive function. The identification of crucial signalling pathways that are related to cancer onset and progression whose master regulators interacts with EGCG may disclose intriguing pharmacological targets, and eventually lead to novel combined treatments in which EGCG acts synergistically with known drugs.

## 1. Introduction

Green tea is produced from Camellia sinensis and it represents the second most consumed beverage in the world after water, being used primarily in Asia and in the Middle East [1].

Several observational and intervention studies have demonstrated that green tea consumption has beneficial effects on many human diseases, including obesity, metabolic syndrome, neurodegenerative disorders inflammatory diseases, and cancer [2,3,4,5,6]. The major polyphenolic component of dried green tea extracts is epigallocatechin-gallate (EGCG) EGCG is the most abundant and biologically active catechin from green tea, accounting for at least 50% of the total catechin content in green tea leaves [7]. The biological effects of green tea were initially ascribed to pro- or anti-oxidative properties of catechins. Most of the studies have been conducted administrating green tea extracts or pure EGCG. A typical weakness of many studies is related to data collected in vitro and cell culture systems, following the administration of doses of green tea extracts (or EGCG) much higher than those that were reached in human plasma after green tea consumption. In vivo, administration of the equivalent of two or three cups of green tea leads to a peak in the plasma levels of tea catechins in the sub-micromolar range in humans [8,9].

Several in vitro, in vivo, and clinical studies have shown multiple EGCG anticancer actions. Among them there are anti-proliferative, pro-apoptotic, anti-angiogenic, and anti-invasive functions [10]. Furthermore, EGCG has been observed to impair other processes that are involved in carcinogenesis as inflammation, oxidative stress and hypoxia and to target tumor microenviroment components (e.g., cancer stem cells, fibroblasts, macrophages, and microvasculature) [11]. In several in vitro and in vivo cancer types, EGCG has been shown to act synergistically with other natural compounds (e.g., curcumin, ascorbic acid, quercetin, genestein, caffeine) [10,12] and it has also been testing in combination with currently used chemotherapeutic drugs (e.g., doxorubicin, cisplatin, sunitinib) [13,14,15,16]. Furthermore, in order to improve EGCG bioavailability and stability, novel formulations of the catechin encapsulated in nanoparticles have been developed [17,18,19,20].

Even if the anti-tumoural effect of green tea catechins (and specifically EGCG) has been extensively demonstrated in vitro, their molecular and cellular mechanisms are not yet completely understood [21,22]. 

The anti-cancer effect of EGCG and green tea extracts is mediated through several mechanisms, including stimulation of anti-oxidant activity and activation of detoxification system [23,24], alteration of the cell cycle [25], suppression of mitogen-activated protein kinase (MAPK) and receptor protein kinase (RTKs) pathways [26,27], inhibition of clonal expansion of the tumour-initiating stem cell population [28], and production of epigenetic changes in gene expression [29]. These mechanisms (reviewed recently in [30,31,32]) are not completely understood yet. Green tea catechins are thought to function both as powerful radical scavengers, in particular, under increased oxidative stress conditions [33], and as ROS generators leading to the inhibition of cancer cell growth through the induction of apoptotis [24,34]. Moreover, they have been shown to induce apoptosis in several ways, such as modulating pro- and anti-apoptotic protein (Bax, Bcl-2, Bcl-XL) and cell cycle regulator proteins (cyclins, CDKs) [35]. Green tea catechins are also able to target genes and proteins that are associated with cell proliferation and apoptosis, including RTKs (receptor tyrosine kinases). Several studies described the inhibitory effect of green tea catechins on these receptors and on Ras/extracellular signal-regulated kinase (ERK)/MAPK and phosphatidylinositol 3-kinase (PI3K)/Akt, which are RTKs-related downstream pathways that are often constitutively activated in tumor cells. EGCG negatively modulates the expression of various transcription factors, including Sp1, AP-1, and NF-kB preventing cancer formation [36,37]. Another mechanism that can explain the pleiotropic effects exerted by green tea catechins is represented by the epigenetic changes in gene expression and chromatin organization. The major epigenetic mechanisms are DNA methylation, histone modifications, and expression of noncoding regulatory micro RNA (miRNAs). Green tea catechins can induce an epigenetic reactivation of genes silenced during carcinogenesis or an epigenetic downregulation of oncogenes through the inhibition of DNA methyltransferases (DNMTs) or histone deacetylases (HDACs) activity and the reduction of their expression [38,39].

Micromolar concentrations of EGCG have been shown to exert a wide array of different effects in a cancer cell. The current understanding is that catechins may either interact with a single critical regulator affecting the activity of key enzymes that are involved in important pathways, or by hitting multiple targets in parallel, thereby modulating different pathways simultaneously [40,41]. 

First it is necessary to identify proteins that bind to catechins with high affinity, which may represent the master regulators controlling one or multiple pathways. Using in vitro models, several research teams have identified proteins that are targeted by EGCG. Among these are vimentin, Fyn, ZAP70, insulin-like growth factor 1 receptor, and glucose regulated protein 78 kDa [42,43,44,45,46]. However, the functional effect of green tea catechins on the target protein activity has been demonstrated only at much higher concentrations of EGCG than Kd values, probably because of the non-specific binding of EGCG to other proteins competing for the target [47]. In Table 1 are listed the principal EGCG molecular targets that were identified in cancer cells.

We think that EGCG-protein binding can be important for the beneficial effect of green tea catechins. Green tea catechins bind to a plethora of proteins and the process of the interaction is highly dependent on the folding status and on the conformational properties of the target protein. In this review, we decided to take into consideration only few proteins that, after direct binding to EGCG, alter and affect their downstream pathways promoting anti-cancer effects. These data could be used for a rational drug design of green tea catechins derivatives exploitable for more specific and effective anti-cancer therapies. We will focus particularly on the onset and progression of cancer, describing and discussing the possible molecular mechanisms through which catechins exert their action.

## 2. 67-kDa Laminin Receptor Signalling Pathways

One of the most interesting targets of EGCG action is the 67-kDa laminin receptor (67LR), a non-integrin cell surface receptor whose expression has been shown to be increased in several cancers, such as blood, prostate, breast, gastric, and colon [101,102,103,104,105,106]. The receptor expression is usually correlated with drug resistance, and it contributes positively to cancer cells viability, tumour progression, metastatic diffusion, and neo-angiogenesis [101,102,107,108].

In 2004, Tachibana et al. identified for the first time the 67LR as a specific EGCG membrane receptor using surface plasmon resonance. The study revealed that 67LR was able to bind EGCG with a Kd value of 39.9 nM. This interaction enabled EGCG to reduce the growth of the lung cancer cell line A549 [90], thus exerting anticancer activity. Other green tea components, such as caffeine, quercetin, epicatechin (EC), and epigallocatechin (EGC) were tested for binding to 67LR, but none were specific ligands of the receptor or showed tumour suppressive effects [90]. Therefore, EGCG appears to be the only catechin able to bind 67LR. Subsequently, a putative EGCG binding site corresponding to the region between the residues 161 and 170 of the receptor has been identified [109]. The direct binding between EGCG and 67LR has been confirmed in prostate cancer cells by Yu et al. [104]. Using MVD (Molegro Virtual Docker, an integrated platform for predicting protein ligand interactions), these authors identified a binding site for EGCG with the same sequence of the laminin tyrosine-isoleucine-glycine-serine-arginine (YIGSR) peptide, corresponding to the 929–933 sequence of β1 chain of 67LR [104].

Many studies have explored the signalling cascades that are triggered by EGCG-67LR interaction, some of which will be discussed in this review. In many cases, the tumour suppression pathway affected ordered microdomains of the cell membrane known as lipid rafts, where the 67LR has been located [110]. Lipid rafts differ from the surrounding membrane, because their composition is enriched in specific lipids (sphingomyelins and glycosphingolipids) and cholesterol, which are tightly packed to form liquid ordered assemblies [111]. Lipid rafts are dynamic, heterogeneous structures whose composition is extremely variable, not only in relation to the lipid and sterols content, but also because of the several proteins that can be recruited (e.g., BCR, FcεRI) or harboured (e.g., Scr tyrosin kinases) [112,113,114,115]. Lipid rafts are rich in tyrosine kinase receptors (RTKs), such as EGFR [116,117,118], IGF1R [119], and HER2 [120]. These receptors have been found to be inhibited by EGCG in several in vitro and in vivo cancer models (e.g., colon, lung, liver and breast cancers) [92,121,122,123]. The functional proteins recruited by lipid rafts allow these structures to play complex roles. Lipid rafts can float within the plasma membrane or can cluster in larger and stabilized platforms in response to different stimuli. In most cases, the lipid rafts clustering allows for the activation of the proteins [124]. Furthermore, modifications in lipid rafts/protein interaction can lead to alterations in lipid and sterol content, which can, in turn, influence lipid raft functions. Thanks to their capability to interact with several cellular and molecular factors as caveolae [111], viruses [125], bacteria, inflammatory molecules [126,127], and growth factors [128,129,130], these microdomains are involved in a plethora of biological functions, like cell polarization, membrane trafficking [111], pathogen internalization [126], and regulation of a wide spectrum of signal transduction pathways [131]. Because most of these pathways can control cancer development, progression, rate of cell proliferation [114], migration, invasion [132,133], and apoptosis [134], lipid rafts composition and functions have received much attention. In addition, many anti-cancer agents (e.g., edelfosine, avicin D, resveratrol) exert their anti-tumour activity, at least in part, by altering or disrupting the structure of lipid rafts [135,136]. 

EGCG has been found to bind to the plasma membrane by interacting with the lipid rafts. The first evidence of this association was shown in the basophilic cell line KU812, where the suppressive action of EGCG on the expression of the high-affinity immunoglobulin E receptor (FcεRI) was triggered by direct binding to lipid rafts. This was mediated by the inhibition of Erk1/2 kinases phosphorylation and activation [51]. Shortly after, the same research team observed that the down-regulation of FcεRI was driven by EGCG through binding to 67LR, a receptor associated with lipid rafts [110]. Others reported that the EGCG inhibitory effect on EGFR in colon cancer cell line HT29 [92], and on HGFR in prostate cancer cell line DU145 [93] was mediated by the alteration of lipid rafts. The collection of signalling pathways affected by lipid rafts structure/function via EGCG/67LR quickly increased in number, as reported in several tumour models, such as multiple myeloma, mammary and epidermiod carcinoma, and chronic myeloid leukemia [52,137,138,139].

### 2.1. Lipid Rafts-Mediated Apoptosis

#### 2.1.1. EGCG/67LR/Akt/eNOS/NO/cGMP/PKCδ/aSMase Pathway

**Figure 1:**
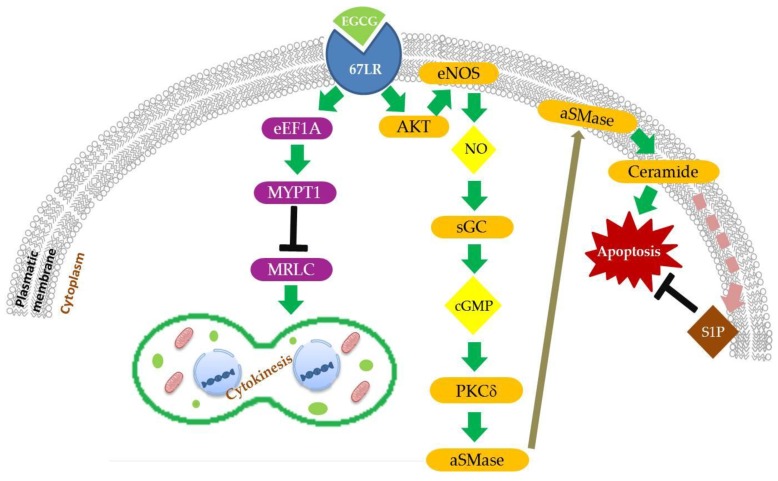
EGCG modulates cell division and apoptosis via 67LR. EGCG binding to 67-kDa laminin receptor (67LR) activates apoptosis program through enhanced nitric oxide (NO) and cGMP production, acid sphingomyelinase (αSMase) activation and ceramide generation. Ceramide metabolization in sphingosine-1-phosphate (S1P) reduces cell apoptosis. EGCG binding to 67LR inhibits via eukaryotic translation elongation factor 1a (eEF1A) cell cytokinesis inducing myosin phosphatase target subunit (MYPT1) dephosphorylation and activation and myosin II regulatory light chain (MRLC) dephosphorylation and inactivation.

Together with the inhibition of cell proliferation, migration, and angiogenesis, induction of apoptosis is one of the main mechanisms through which EGCG exerts its anti-tumour activity [140,141]. Several studies reported that EGCG is able to affect the expression and function of anti-apoptotic factors (e.g., Bcl-2, Bcl-xl) and to up-regulate pro-apoptotic molecules (e.g., Bax, caspase-3) in several cancer models [58,142,143,144]. However, the mechanisms through which EGCG modulates key cell death regulators are not completely understood. Some studies reported that 67LR plays a relevant role in triggering apoptosis after binding its ligand, EGCG, in haematological malignancies, such as acute myeloid leukemia and multiple myeloma [145,146]. More recently, a signalling pathway inducing EGCG/67LR-dependent apoptosis through the activation of protein kinase Cδ (PKCδ), acid sphingomyelinase (aSMase), and lipid rafts clustering has been described in multiple myeloma models [137] (Figure 1). The enzyme aSMase is responsible for the catabolism of sphingomyelin (SM) and is known to be part of the signalling cascades that mediates lipid raft-dependent apoptosis [147]. It can be activated in response to external pro-apoptotic stimuli as physical agents (e.g., radiation, UVA light) [148,149], anti-cancer drugs (e.g., cisplantin, doxorubicin) [150,151], and pro-apoptotic receptors (e.g., Fas, TNF-R) [147,152]. One of the best described mechanisms of aSMase activation is triggered by Fas receptor. The binding between death receptor Fas, harboured in lipid rafts [153], and its ligand FasL lead to the recruitment of adaptor Fas-associated protein with death domain FADD, which in turn recruits and activates pro-caspase 8. The final death-inducing signalling complex (DISC) then activates aSMase, which migrates from the cytoplasmic compartment to lipid rafts, where it generates the sphingolipid ceramide from SM [154,155,156]. In response to ceramide generation, cholesterol is displaced from lipid rafts, thus leading to an increase of membrane fluidity [157]. Ceramide plays a role as second messenger in the signal transduction, inducing lipid raft clustering and the stabilization of DISC complex, amplification of Fas/FasL signalling, finally leading to apoptosis [155,158,159]. A similar mechanism has been hypothesized in the case of cervical, prostate and colon cancer, where EGCG administration induces cell apoptosis through aSMase activation and ceramide increase [160,161,162]. 

Studies on multiple myeloma cell lines in vitro and in vivo, in patients or murine models, showed that the activation of 67LR through EGCG binding induces the activation of PKCδ after phosphorylation of Ser664. Activation of PKCδ leads, in turn, to aSMase activation, and finally to cell apoptosis [137]. These authors pointed out that treatment with 5 µM EGCG led to an increase of nitric oxide (NO) [74]. NO is an inorganic signalling messenger triggering a wide range of cellular pathways. The increase in NO levels is due to the activation of endothelial nitric oxide synthase (eNOS), after phosphorylation in the residue Ser1177 by Akt kinase [74]. Production of NO causes an increase of cGMP, produced by NO-dependent soluble guanylate cyclase (sGC) activation, and then the phosphorylation of PKCδ [74] (Figure 1). Furthermore, more recently, it has been observed that the anticancer agent coptisine induces apoptosis in hepatocellular carcinoma (HCC) cells via the 67LR/cGMP pathway [163]. Conversely, several studies reported that EGCG negatively regulates eNOS/NO production in different cancer types [75,76,77,78] and also sGC/cGMP amount [96]. 

However, the fact that administration of 5 µM EGCG was sufficient to enhance NO production, but not a significant increase of cGMP levels to induce cell apoptosis, gives rise to the question of whether other factors might interfere with cGMP-mediated aSMase activation (Figure 1). Enzyme phosphodiesterase 5 (PDE5), one of the major cGMP negative regulators, was found to be highly expressed in multiple myeloma patients as compared to healthy donors, suggesting that PDE5 could be a target for a possible combinatorial therapy with 5 µM EGCG [74]. This experimental approach has been implemented. The combined treatment of PDE5 inhibitor Vardenafil and 5 µM EGCG caused a strong reduction of cell viability not only in multiple myeloma, but also in other models as prostate, gastric, pancreatic, breast cancer, and in acute myeloid and chronic lymphocytic leukemia cell lines [74,164,165]. Vardenafil and EGCG synergistic action has been found to cause a significant reduction of IC_50_ of EGCG [74,164,165]. The tumour suppressive effects of the combinatorial therapy have also been confirmed in vivo in xenograft murine models of multiple myeloma, treatment that resulted in the reduction of tumour volume and increased survival without hepatotoxicity, a possible side effect of high EGCG administration [74]. In this model, controls (namely cell lines and primary cultures, as well as healthy animal models), were not affected by EGCG alone or in combination with Vardenafil.

#### 2.1.2. EGCG/67LR/Ceramide/SphK1/S1P Pathway

The formation of larger platforms of cholesterol-enriched lipid rafts in cancer cells is often associated with aberrant activation of RTKs, resulting in increased proliferation, survival, and metastatic spread [166]. Instead, ceramide causes cholesterol displacement from lipid rafts, formation of ceramide-enriched lipid rafts, and induction of cell apoptosis [147]. Therefore, ceramide catabolism/degradation may produce anti-apoptotic effects. Ceramide can be deacetylazed and converted to sphingosine, which can be phosphorylated to sphingosine-1-phosphate (S1P) by the sphingosine kinase 1 (SphK1), an enzyme that is highly expressed in several cancers. The S1P can activate protein G-coupled receptors that can in turn activate pro-survival and anti-apoptotic signalling. In prostate cancer models, treatment with high doses of EGCG (75 µM) suppressed tumour growth in vitro and in vivo through the inhibition of SphK1/S1P signalling [167] (Figure 1). 

The lesson from these data is that, in a particular cell system, a correct balance between ceramide synthesis and catabolism is fundamental [168,169]. Treatment with 1 µM and 5 µM EGCG in the multiple myeloma cell line U266 caused the induction of aSMase activity [52]. However, ceramide accumulation has been observed only after giving high concentrations of EGCG (10 µM and 20 µM EGCG) [52]. Treatment with these high doses of EGCG leads to the disruption of cholesterol-enriched lipid rafts and the inhibition of phosphorylation and the activation of several RTKs (e.g., EGFR, ErbB2, ErbB3, HGFR, IGF1R, Mer, and Flt3). IGFR inhibition has been demonstrated to be dependent on 67LR and aSMase expression [52]. Because the amount of SphK1 has been found to be increased in multiple myeloma cell lines and specimens from patients, the combinatorial treatment of 5 µM EGCG and SphK1 inhibitor Safingol was tested. The data demonstrate that the combination of the two drugs caused an increase in ceramide content, the disruption of cholesterol-enriched lipid rafts, inhibition of RTKs phosphorylation, and finally an increase of cell apoptosis [52]. Furthermore, the combination treatment also affected another cell death mediator that was activated by ceramide, the death-associated protein kinase 1 (DAPK1), causing the de-phosphorylation of DAPK1 inhibitory residue Ser308 and leading to its activation [52]. 

Thus, like the inhibition of PDE5 with Vardenafil, the simultaneous action on two related pathways employing two agents in combination, produced a synergistic effect that strongly reduced the IC_50_ of EGCG [52]. The onco-suppressive action of the double treatment (EGCG plus Safingol) has been found effective in vitro in acute myeloid leukemia, chronic myeloid leukemia, and in chronic lymphocytic leukemia models [170]. Absence of toxicity of the combined therapy has also been shown in vivo [52].

### 2.2. Cancer Cell Growth Inhibition

#### 2.2.1. EGCG/67LR/eEF1a/MYPT1/MRLC Pathway

EGCG can exert anticancer functions inducing cell cycle arrest. Several studies reported the blockade of the cell division cycle by EGCG administration in G0, G1, S, and G2 phases. EGCG may act through the indirect downregulation of pro-proliferative factors, such as cyclin D1, cyclin E, cyclin A, cyclin B, CDK4, CDK6, CDK2, and CDK1, as well as by the upregulation of anti-proliferative effectors, such as CDK inhibitors p27, p21, p16, and p18 [35,48,56,66,171,172]. In addition, EGCG has been found to act on cytokinesis, a critic step of cell division, by interacting with 67LR receptor [55,61,173]. 

Cytokinesis is the final step of cell division, leading a mother cell to be divided into two daughter cells. Early events of the process require the formation of an actomyosin ring, also known as contractile ring, that allows the formation of the cleavage furrow at the equator of mitotic cells [174,175]. Generation of the furrow enables the equal division of genetic material between the two forming cells and their subsequent separation. The interaction between actin filaments (F-actin) and myosin motors is controlled by different processes among which is the phosphorylation/dephosphorylation of the myosin II regulatory light chain (MRLC). Myosin II is one of the main motors involved in cytokinesis, activated through MRLC phosphorylation at Ser19/Thr18 by kinases, such as MLCK, ROCK, and Citron kinase [176]. Ser19 phosphorylation favours the interaction with F-actin, the contractile ring formation, and filaments assembly. A di-phosphorylation seems to be involved in the assembly of filaments, but the role of phosphorylation in Thr18 alone is less clear [177,178,179]. Conversely, MRLC dephosphorylation in Ser19 or Ser19/Thr18 by the myosin phosphatase leads to myosin inactivation. The MRLC activity is also indirectly regulated through the phosphorylation of myosin phosphatase itself. When the largest region of myosin phosphatase, called myosin phosphatase target subunit (MYPT1), is phosphorylated in at least one of the inhibitory sites (e.g., Thr696, Thr853), its activity is inhibited, and, as a consequence, MRLC remains active, thus providing a positive signal triggering cytokinesis [180]. 

EGCG has been found to be able to interfere with the cytokinesis of HeLa cells through its action on MRLC phosphorylation status, thereby affecting the cellular growth [55]. EGCG activates the signalling cascade that is responsible for the impaired MRLC phosphorylation through binding to its membrane receptor 67LR [55] (Figure 1). At first, it was reported that the treatment of HeLa cells with 10, 20, and 50 µM EGCG resulted in the disruption of stress fibers, reduction of the contractile ring formation, increment of cells blocked in G2/M phases, and inhibition of cell growth [55]. Further analyses revealed that EGCG treatment also caused, via 67LR, a reduction in single Ser19 and in double Ser19/Thr18 MRLC phosphorylation, which effects on MRLC phosphorylation might reasonably trigger the effects shown on cell division and growth [55]. Under similar conditions, EGCG was also found to decrease the phosphorylation of MYTP1 at inhibitory site Thr696 both in vitro and in vivo, thus preventing myosin phosphatase inactivation. According to the literature, the ability of EGCG to interfere with MRLC phosphorylation could be the indirect consequence of MYPT1 loss of inhibition [55]. Recently, another factor has been added to the members of the EGCG signalling pathway, believed to be responsible for impaired cancer cell cytokinesis: the eukaryotic translation elongation factor 1a (eEF1a), which has been found to be necessary to enable EGCG to alter MYPT1 phosphorylation status [61]. eEF1a is mainly known as a component of the eukaryotic translation machinery, but it also takes part in other cellular processes, such as senescence, oncogenic transformation, and cell proliferation [181,182,183]. eEF1a is able to bind to MYPT1 and F-actin [184]. In vitro and in vivo experiments demonstrated that no significant reduction in MYPT1 and MRLC phosphorylation, actin disassembly and cell proliferation was observed after EGCG administration in eEF1a knockout models [61]. This evidence has been further corroborated by the observation that when eEF1a levels are restored and 67LR is absent, the effects that are described above disappear as well. Thereby, eEF1a is thought to be downstream of 67LR and upstream of MYPT1 in the signalling pathway that is triggered by EGCG [61] (Figure 1). 

#### 2.2.2. EGCG/67LR/cAMP/PKA/PP2A Pathway

67LR surface receptor is involved in the selective anti-tumour activity exerted by EGCG in melanomas. Tsukamoto et al. identified protein phosphatase 2A (PP2A) as a downstream target of 67LR in melanoma cells [86]. PP2A is a Ser/Thr phosphatase that is involved in important cellular processes, such as proliferation, signal transduction, and apoptosis, and it is considered to be a tumor suppressor that is functionally inactivated in cancer [185,186].

By performing functional genetic screening, Tsukamoto and colleagues showed that EGCG binding to 67LR receptor induces PP2A activation mediated by the cAMP/PKA pathway [86], which led to the suppression of melanoma tumor cell growth. Even though the direct interaction between EGCG and PP2A was demonstrated using very high EGCG concentrations [84,187,188], 1 µM EGCG was sufficient to activate 67LR/PP2A pathway. PP2A directly interacts with p70S6k and down-regulates mTOR signaling [189], which is usually aberrantly activated in melanomas. Therefore, it represents an important contribution to chemotherapeutic resistance of commonly used BRAF inhibitor treatment. The EGCG-activating 67LR/PP2A pathway exerts a strong synergistic effect with PLX4720, a BRAF inhibitor, in drug-resistant melanomas.

Another effect that is mediated by the 67LR/PP2A signaling is the activation of Merlin, a tumor suppressor protein that is encoded by the NF2 gene at physiological concentrations of EGCG, as low as 1 µM [86]. Merlin activity seems to target cell surface RTKs and adhesion/extracellular matrix receptors, regulating cell proliferation, survival and motility [190]. PKA, p21-activated kinase 1 and 2 (PAK 1/2), or MYPT can activate Merlin by dephosphorylation at Ser-518. In the study by Tsukamoto et al. [86], EGCG was demonstrated to be an activator of Merlin via 67LR/PP2A pathway. In prostate cancer cell lines the absence or inactivation of Merlin contributes to tumor development and progression toward a highly invasive and chemo-resistant state [191,192,193].

Recently published data show that 10 µM EGCG up-regulates let-7b miRNA expression not only in melanoma cell lines, but also in metastatic melanoma tumours in vivo [81]. miRNAs are non-coding RNAs transcripts that are able to regulate fundamental biological activities related to mRNA degradation or translational inhibition [194]. Yamada et al. demonstrated that 67LR is involved in the EGCG-elicited let-7b increase, which leads to the inhibition of melanoma tumor progression [81]. Let-7b recognizes multiple target genes that are related to tumor progression, such as the high mobility group A2 (HMGA2), decreased in EGCG-treated melanoma cells [81], or Ras [195,196].

Furthermore, the data indicated that PP2A inactivation caused the induction of let-7b, which is generally down-regulated in cancer (including melanoma and prostate cancer) [81,197], even if it is not clear whether let-7b transcription or let-7b processing is modulated by EGCG-induced PP2A activation.

Zhou et al. confirmed that EGCG induced miRNAs profile changes in a mouse model of lung tumor. They highlighted that the miRNAs affected by EGCG and target genes are different from those that were previously identified by in vivo studies [198].

### 2.3. Modulation of Cancer Stem Cells Properties

EGCG was shown to affect the survival of cancer stem cells (CSCs). EGCG inhibits CSCs growth and stemness in several malignancies, such as breast [199], lung [54,200], colorectal cancer [85], osteosarcoma [14], and neuroblastoma [201].

Kumazoe M. et al. [202] describe the effects of EGCG on the features of pancreatic CSCs (i.e., the capability to form colonies and spheroids) through the activation of the EGCG/67LR/cGMP axis. The same research team had observed that spheroid formation in pancreatic CSCs colonies was inhibited by cGMP targeting of the Forkhead box O3 (FOXO3)/CD44 axes [203]. Transcriptional factor FOXO3 is known to be a cancer suppressor, but it also induces the high expression of CD44, a master regulator (and also a marker) of CSCs [202]. FOXO3 has been shown to be a direct target of EGCG in tumours, like pancreatic and breast cancer. In pancreatic cancer treatment with EGCG suppressed tumour growth, accompanied by FOXO3 downregulation [99]. By contrast, in breast cancer, a positive regulation of FOXO3 exerted by the EGCG has been described [70,204,205]. Although the reported modulations seem to be opposite, the action of EGCG on FOXO3 seems to lead to cancer suppression altogether. Recently, the role of EGCG in inhibiting cancer stem cells (CSC) growth and altering their features is emerging [54,95,199]. According to this literature, EGCG seems to act by downregulating CD44 expression in tumours, like non-small cell lung cancer and pancreatic cancer [200,202]. In pancreatic cancer cell lines expressing CD44, the isoform 3A of the enzyme phosphodiesterase (PDE3A) is highly expressed [202]. Like other members of the same family, PDE3A is a negative regulator of cGMP [206]. In pancreatic cancer cells, low EGCG administration did not lead to a significant increase in cGMP amount, or to the reduction of colony and spheroid formation [202]. Further experiments were conducted using low doses of EGCG combined with the administration of a PDE3A inhibitor, Trequinsin. The combination therapy decreased the protein levels of FOXO3 and CD44, caused an increase of cGMP, and a strong reduction in the CSCs capability to form both colonies and spheroids. The combination of EGCG and Trequinsin is synergistic and it reduces the IC_50_ of EGCG, thus allowing for its use at physiological concentration. These observations have also been confirmed in vivo [202]. Surprisingly, as for the other signalling pathway that is discussed above, the effects of EGCG alone, or in combination with other agents, are always specific for cancer cells, and they do not affect normal cells. This highly specific effect of EGCG is still waiting for an explanation.

## 3. Other EGCG-Interacting Proteins

Another interesting protein that was shown to interact directly with green tea catechins is the human peptidyl prolyl *cis*/*trans* isomerase (Pin1). Pin is a protein with two domains: an N-terminal WW-domain and a C-terminal PPIase domain; both are necessary for its function. Although many PPIases have been identified some with an established role in cancer, only Pin1 acts distinctively and specifically on phosphorylated proteins. Pin1 catalyzes the *cis*/*trans* isomerization of the peptidyl proline bond of proteins. This activity causes major changes in the conformation of the target protein, with a consequent alteration of its function or stability. In this way, Pin1 affects and modulatse different pathways involving kinase-dependent signaling, such as NF-kB, activator-protein 1 (AP-1), nuclear factor of activated T cells (NFAT), or b-catenin [207]. Pin1 has been demonstrated to have a major role in oncogenic signaling [208,209] and is highly expressed in several cancers [210,211], including prostate cancer [212].

Urusova et al. used crystallographic and biochemical data to show that EGCG interacts directly with both the PPase and WW domains of Pin1, which inhibits its tumour-promoting activity. Therefore, Pin1 represent a possible target for anti-cancer therapies [83,213]. The dissociation constant of EGCG and Pin1 has been calculated as 21 µM, both by protease-coupled and isothermal titration calorimetric assays: this value is similar to the concentration of EGCG that was found to exert anti-cancer effects in experimental cancer models [40]. Since the Kd value that resulted was quite high, the interaction between EGCG and Pin1 was described as “not strong”. Urusova and colleagues crystallized the Pin1-EGCG complex, resolving its structure at 1.9 Å resolution by X-ray diffraction. The crystal structure has revealed that a molecule of EGCG was bound to Pin1 WW domain (aminoacids 1–31), which is responsible for the interaction with the substrate, while another molecule of EGCG was bound to the Pin1 PPIase domain, necessary for the isomerization reaction. A recent study demonstrated that galloyl group in EGCG is required for Pin1 inhibition [214]. Binding between EGCG and Pin1 in solution has been studied recently by combining fluorescence spectrum, far-UV circular dichroism spectrum with molecular dynamics simulations. The analysis of the binding energy confirmed the strong inhibitory effect that is exerted by EGCG on Pin1 activity [215].

To analyze the functional consequence of Pin1-EGCG binding, Urusova and colleagues used mouse embryonal fibroblasts (MEF) collected from PIN1 KO and WT mice, and showed that Pin1 expression is required for EGCG (10–40 µM) inhibitory effect on MEFs growth. Furthermore, the formation of the EGCG-Pin1 complex prevented the binding of the Pin1 substrate c-Jun. Finally, EGCG effect on transcriptional regulation of AP-1 and NF-kB has been shown to be mediated by Pin1 [83].

Green tea catechins are mainly believed to prevent cancer. However, several epidemiological studies suggest that their activity also works against cancer progression; the interaction of EGCG with proteins that are involved in cancer progression and metastatic spread has been considered. One of the effects exerted by EGCG is the inhibition of TGF-β signaling transduction. TGF-β is a multifunctional cytokine that induces epithelial-mesenchymal transition (EMT) of cancer cells, and it is also responsible for the maintenance of EMT, a critical event during early metastatic growth. The mechanism by which EGCG modulates TGF-β pathway has not been completely elucidated. It has been shown that the binding between TGF-β and its receptor, TGFR-II, activates two different pathways leading to EMT: the canonical Smad-dependent pathway and the mitogen-activated protein kinase (MAPK) pathway. Tabuchi et al. used immunoprecipitation and affinity chromatography assays to demonstrate binding between EGCG and TGFR-II protein. This interaction may be responsible for the inhibitory effect of EGCG on the expression of alpha-SMA (considered a marker of the EMT) via the TGF-beta Smad2/3 pathway in human lung fibroblast cells [94].

EGCG has also been shown to bind to metalloproteinases (MMPs). MMPs are matrix degrading enzymes that are involved in tumor invasion and metastasis [50] whose expression is regulated by several growth factors, including TGF-β1 [216,217,218,219]. Sazuka et al. have demonstrated that EGCG inhibits the collagenase activity of MMP-2 and MMP-9 produced by lung carcinoma cells. The authors suggest that the mechanism of inhibition relies on direct binding between EGCG and MMP proteins, as proved by affinity gel chromatography experiments [50]. In 2017, Chowdhury et al. performed a preliminary in silico analysis and then showed a strong interaction of pro-/active MMP2 with the galloyl group of EGCG and ECG in pulmonary artery smooth muscle cell culture supernatant. They showed that EGCG and ECG were better inhibitors of proMMP2 when compared to MMP2, and they demonstrated that a strong interaction with MT1/MMP is involved in the conversion of proMMP2 to active MMP2 [220]. Further, investigating the interactions of pro-/active MMP-9 with green tea catechins by computational methods, they showed strong interactions between pro-/active MMP9 and EGCG/ECG [221].

## 4. EGCG Epigenetic Regulation

Another mechanism that can explain the pleiotropic effects exerted by green tea catechins in tumor cells is the epigenetic change in gene expression and chromatin organization. Mutations in oncogenes and tumor suppressor genes are often the cause of cancer development and alterations of gene expression count for cancer progression. 

Many biologically active compounds, including EGCG, have been demonstrated to modulate DNA methylation and histone acetylation status [222].

DNA methyltransferases (DNMTs) and histone deacetylases (HDACs) are enzymes that are involved in transcriptional gene silencing and histone acetyl transferases (HATs) positively regulate gene expression regulation [223,224]. Several studies reported EGCG contribution in epigenetic control acting on DNMTs, HDACs and HATs expression and activity in different tumours. We will briefly mention different genes whose expression is enhanced or reduced by EGCG-dependent epigenetic control. 

Fang et al. demonstrated that EGCG binds to DNMT and competitively inhibits the enzymatic activity (Ki of 6.89 µM), yielding the reactivation of methylation-silenced genes in prostate cancer PC3 cells [225]. Molecular modeling and docking studies supported the binding of EGCG to DNMT3B and HDAC1 [39].

In HeLa cell line, it has been observed that EGCG can direct bind to and inhibit DNMT1, DNMT3B, and HDAC1 activity, causing a reduction in DNA hypermethylation and restoring the expression of repressed genes as retinoic acid receptor β (RARβ), CDH1 (e-cadherine gene), and DAPK1 [29,39]. Furthermore, in the same the same cell line, EGCG combination with eugenol-amrogentin (active compounds of clove and Swertia Chirata, respectively) reduces DNMT1 expression with the consequent hypomethylation of the cell cycle inhibitors p16 and LimD1 promoters [226]. In acute promyelocytic leukemia cells, EGCG down-regulates DNMT1, HDAC1, HDAC2, G9a, and Polycomb repressive complex 2 (PRC2) core components expression and favours the binding of hyperacetylated H4 and acetylated H3K14 histones to promoter regions of p27, CAF, C/EBPα, and C/EBPε genes [63]. In the lung cell line PC-9, EGCG combination with Am80 (a synthetic retinoid used for acute promyelocytic leukemia therapy) causes a decrease of HDAC4, HDAC5, and HDAC6 protein levels and reduction of HDAC activity, leading to increased p53 and α-tubulin acetylation [227]. In in vitro and in vivo models of lung cancer, EGCG has been found to resensitize tumor cells to Cisplatin (DDP)-based combination chemotherapy through DNMT and HDAC activity inhibition, and the subsequent re-expression of GAS1, TIMP4, ICAM1, and WISP2 genes [228]. In in vivo model of lung cancer, EGCG epigenetic action in down-regulating DNMT1 is accompanied by phospho-histone H2AX (γ-H2AX) and p-AKT reduction [229]. In skin cancer cells, it has demonstrated EGCG capability in reducing DNMT1, DNMT3A, and DNMT3B activity and expression, and also in increasing histones H3 and H4 acetylation. As a consequence of the described epigenetic changes, a restored expression of the cell cycle inhibitors p16 and p21 has been observed [48]. In breast cancer cells, EGCG-dependent reduction of HDAC1 and zeste homolog 2 (EZH2) protein levels leads to tissue inhibitor of matrix metalloproteinase-3 (TIMP-3) gene transcriptional activation [72]. In prostate cancer cell lines, it has been observed that the EGCG-dependent reduction of the acetylated androgen receptor (AR) gene might be induced by EGCG reduction in HAT activity [65]. EGCG also acts on teleomerase, reducing its activity in different tumor types as esophageal carcinoma [230], glioma [231], cervical cancer [232], breast cancer [100], nasopharyngeal carcinoma [233], ovarian cancer [68], laryngeal squamous cell carcinoma [234], and lung cancer [235]. It has also been shown that EGCG can translocate from the cytoplasm to the nucleus where it can bind to DNA, suggesting a possible role in gene expression regulation also through the direct binding to nucleic acid [236,237]. However, the effects of EGCG/DNA direct interaction need to be clarified.

## 5. Conclusions

Because of their anti-proliferative, pro-apoptotic, and anti-oxidative properties, green tea catechins and especially EGCG are receiving much attention in cancer biology. Several in vitro, in vivo, and clinical studies, have demonstrated that EGCG exerts anti-cancer effects in different models through the activation/inhibition of several signalling pathways, most of which are triggered by the direct interaction between EGCG and specific protein targets. The array of EGCG interactors is wide and growing, and it includes intracellular molecules, membranes receptors, membrane microdomains, and the plasma membrane itself. One of the first EGCG direct target identified was 67LR, but in recent years, others interactors, such as Pin1 or TGFR-II, have been recognized. Appropriate identification and study of EGCG direct targets will allow a better understanding of its mechanisms of action and a better exploitation of its anti-cancer properties. From 2004, when the 67LR was first identified as direct target of EGCG by Tachibana et al., several research teams have investigated the pathways modulated by EGCG-67LR interaction. Today, we know that the anti-proliferative action of EGCG is mediated by the binding to 67LR, whose expression is increased in tumour cells. Convincing experimental data also showed that membrane composition is involved in the inhibitory activity of EGCG in some cancer cells lines. Since 67LR is generally located in lipid rafts, EGCG-mediated microdomains composition and the alteration of their functions triggers the downstream signalling cascades. In addition, new experimental data have brought to light novel EGCG signalling cascades leading to cell apoptosis, cell cycle arrest, reduction in CSC colony and spheroid formation, as well as regulation of miRNAs expression. EGCG binding to membrane receptors, such as TGFR-II, intracellular molecules, such as Pin1 and secreted enzymes, such as MMPs, provided noteworthy information about the mechanisms of EGCG-mediated tumour suppression. Another mechanism to explain the pleiotropic anti-cancer effects that are exerted by EGCG and green tea catechins that is gaining the attention of the researchers is the modulation of epigenetic processes. Long-term administration of green tea catechins leads to the re-activation of tumour suppressor genes that are silenced during carcinogenesis and downregulation of oncogenes through the inhibition of enzymes, such as DNMTs and HDACs involved in DNA methylation and chromatin remodelling. Further studies on the interaction of EGCG with protein targets will provide new insights enabling the development of more pharmacological treatments targeting EGCG-activated master regulators of key pathways.

## Figures and Tables

**Table 1:** Epigallocatechin-gallate (EGCG) molecular targets that are involved in cancer onset and progression.

Cell Cycle, Proliferation & Survival	Apoptosis & Cell Death	Motility, Invasion and Metastatization	Inflammation	Epigenetic Control	Others
p16 [48]	Bax [49]	MMP-2 * [50]	FcεRI [51]	DNMT1 * [39]	DAPK1 [52]
p18 [35]	Bad [53]	MMP-9 * [50]	IL-8 [54]	DNMT3A [48]	MRLC [55]
p21 [48]	Bak [56]	MMP-14 [57]	IGF-1R * [45]	DNMT3B * [39]	MYPT1 [55]
p27 [56]	Bcl-2 * [58]	uPA [59]	VEGF [60]	HDAC1 * [39]	eEF1a [61]
Cyclin D [56]	Bcl-xl [53]	PAI-1 [59]	CSF-1 [62]	HDAC2 [63]	ID1 [64]
Cyclin E [35]	Bcl-xs [56]	E-cadherine [39]	CCL-2 [62]	HAT [65]	RAR-β [39]
Cyclin A [66]	Caspase3 [56]	SLUG [67]	COX-2 [60]	hTERT [68]	HSP70 [53]
Cyclin B [66]	Caspase8 [69]	SNAIL1 [70]	iNOS [71]	EZH2 [72]	HSP90 * [73]
CDK4 [56]	Caspase9 [56]	Vimentin * [42]	eNOS [74,75,76,77,78]		GRP78 * [46]
CDK6 [56]	Apaf-1 [53]	Twist [79]			PECAM-1 [80]
CDK2 [35]	Puma [56]	N-cadherine [79]			miR-16 [62]
CDK1 [66]	XIAP [53]	HIF-1α [60]			let-7b miRNA [81]
Erk1/2 [56]	Cytochrome C [56]	β-catenin [54]			miR-210 [82]
Pin * [83]	p53 [84]	Wnt [54]			miR34a [85]
PPA2 [86]	Survivin [87]	TIMP-3 [72]			miR145 [85]
PKA [86]	Fas [69]				miR200c [85]
STAT [12]	DR5 [69]				ZAP70 * [44]
AR [65]	PARP [88]				TRAF-6 * [89]
67LR * [90]					Oct4 [85]
FcεRI [51]					Sox2 [91]
EGFR [92]					Notch1 [85]
HGFR [93]					Nanog [85]
TGFR-II * [94]					CD133 [95]
cGMP [74] [96]					
cAMP [86]					
P-glycoprotein [88]					
NF-kB [97]					
c-Myc [98]					
FOXO3a [99]					
GSK-3β [98]					
PI3K [100]					
AKT [100]					
PKC-δ [74]					
JAK-1/2 [12]					
Src [57]					
CK1α [98]					
p38 MAPK [56]					
JNK [56]					

* EGCG direct interactors.
